# Efficient and Pure I-III-VI AIGS Quantum Dot-Based Light-Emitting Diodes via Ligand-Reshaped Surface State

**DOI:** 10.1007/s40820-026-02086-y

**Published:** 2026-02-09

**Authors:** Leimeng Xu, Jianpeng Zhao, Jindi Wang, Jisong Yao, Shalong Wang, Zhi Wu, Jizhong Song

**Affiliations:** Key Laboratory of Materials Physics of Ministry of Education, Laboratory of Zhongyuan Light, School of Physics, Zhengzhou University, Daxue Road 75, Zhengzhou, 450051 People’s Republic of China

**Keywords:** Quantum dots, Silver indium gallium sulfide, QD-based light-emitting diodes, External quantum efficiency, Color purity

## Abstract

The I-III-VI silver indium gallium sulfide (AIGS) quantum dots (QDs) have gained extensive attention owing to their tunable emission wavelength and ecofriendly composition; however, the performance of AIGS QD-based light-emitting diodes (QLEDs) remains constrained by suboptimal surface state, significantly lagging behind that of other heavy-metal-containing QD counterparts. Herein, we propose a ligand-reshaped strategy aimed at optimizing the surface state of AIGS QDs to enhance the performance of QLEDs. A polyfunctional ligand, dimercaptosuccinic acid (DSA), is introduced to reshape the QD surface through passivation of uncoordinated Ga^3+^ and suppression of S vacancies. After DSA passivation, the QDs exhibit not only exceptional luminescent properties with a photoluminescence quantum yield of 89%, but also pure emission with a narrow full width at half maximum of 31 nm. Concurrently, DSA passivation markedly improves the electrical transport characteristic of QDs, thereby ensuring efficient carrier injection. Resultantly, the reshaped QLED achieves a maximum peak external quantum efficiency of 8.4% along with a narrow FWHM of 31 nm, representing a record performance reported thus far for the AIGS system. The proposed DSA ligand-reshaped strategy endows AIGS QLEDs with both high efficiency and color purity, substantially advancing their potential for the application in QD lightings and display technologies.

**Supplementary Information:**

The online version contains supplementary material available at 10.1007/s40820-026-02086-y.

## Highlights

- A ligand reshaped strategy is proposed to optimize the surface state of silver indium gallium sulfide (AIGS) quantum dots (QDs) via a polyfunctional ligand, mercaptosuccinic acid.
- Both donor–acceptor pair and non radiative recombination pathways are decreased after passivation, which leads to a notable narrowing full width at half maximum to 31 nm and an enhancement of photoluminescence quantum yield up to 89%.
- The efficient and pure QD based light emitting diodes (QLEDs) exhibit a maximum peak external quantum efficiency of 8.4%, representing the record performance of AIGS QLEDs.

**Supplementary Information:**

The online version contains supplementary material available at 10.1007/s40820-026-02086-y.

## Graphical abstract

**Figure:**
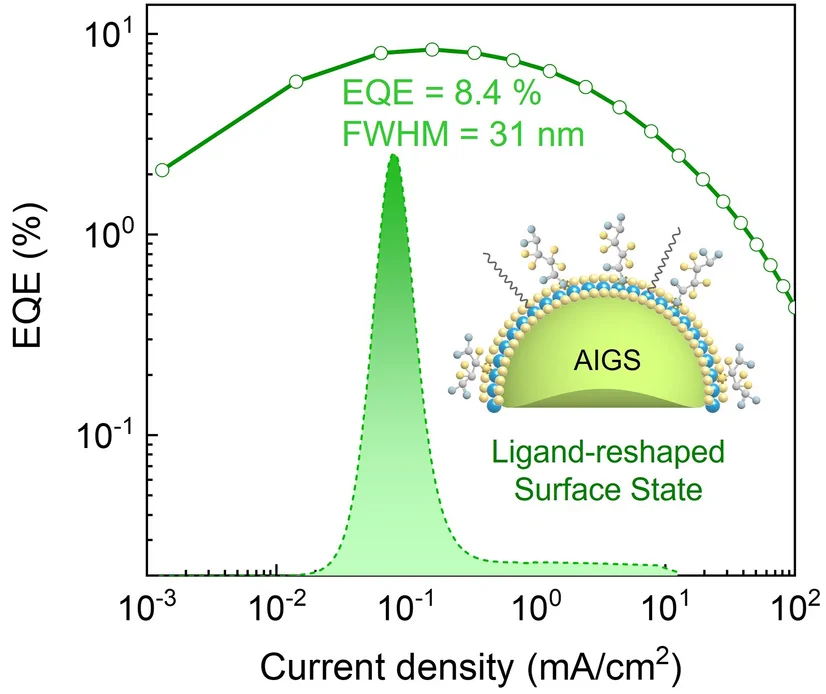

## Introduction

Over recent decades, quantum dot (QD)-based light-emitting diodes (QLEDs) are now capable of commercial competitiveness in high-quality displays due to their exceptional color purity, wide color gamut, and cost-effectiveness [1–5]. Although significant breakthroughs have been made in Cd- and perovskite-based QLEDs, exploring the heavy-metal-free QD counterparts are urgent for the practical implementation of QLED technology. Among potential candidates, I-III-VI alloyed QDs composed of silver indium gallium sulfide (AIGS) have recently attracted considerable attention owing to their environmentally benign composition, direct bandgap, and tunable light-emitting wavelength [6–9]. However, AIGS QDs typically exhibit a broad full width at half maximum (FWHM) exceeding 50 nm, primarily attributed to the luminescence arising from intragap defect state-related band-to-hole and donor–acceptor pair (DAP) recombination mechanisms [10, 11], which has not received extensive attention [12, 13]. Recently, the FWHM of AIGS QDs could be narrowed to approximately 30 nm by enhancing band-to-hole recombination and inhibiting DAP recombination, making the color purity comparable to that of conventional Cd-based and perovskite QDs [6, 14]. These findings highlight the potential of AIGS QDs for the application of high-definition displays and high-quality lighting sources.

Notably, recent studies have reported that AIGS QDs can achieve exceptionally high luminescent efficiency with photoluminescence quantum yield (PLQY) approaching nearly 100% [6, 15]. For instance, Lee et al. enhanced the PLQY of AIGS QDs to 96% through Ag-Ga-S (AGS) coating [6]. Yan et al. introduced Z-type ligands ZnX_2_ to passivate surface defects, which improve the PLQY of AIGS QDs from 28.5 to 87% [16]. Kim et al. suppressed defect-related luminescence and enhanced band-edge emission by modifying Ga halides during the synthesis process, increasing the PLQY of AIGS QDs to 95% [15]. These findings demonstrate that AIGS QDs are promising candidates for the fabrication of highly efficient electroluminescent (EL) devices. However, the currently reported LEDs based on AIGS QDs generally exhibit extremely low external quantum efficiency (EQE), typically around 1%. Although EQE values up to 5% have been achieved by optimizing the device architecture and balancing carrier transport [14], it is still far lower than that of traditional Cd-based and perovskite QDs. To realize high-performance electroluminescence, it is essential to utilize high-quality QDs that possess both excellent PL properties and favorable electrical transport characteristics. Although significant progress has been made in enhancing the PL efficiency of AIGS QDs, the PLQY of the AIGS QDs currently used in devices remains relatively low, typically below 80%. Moreover, the commonly used ligands in AIGS QDs, primarily oleylamine, possess long alkyl chains that exhibit insulating characteristics, which hinders carrier injection and transport, resulting in suboptimal device performance. Consequently, the development of appropriate ligands that enable AIGS QDs to simultaneously achieve high PL feature and effective charge transport is crucial for improving the overall performance of AIGS QLEDs.

In this work, we propose a ligand-reshaping strategy for the successful synthesis of high-quality AIGS QDs that exhibit simultaneously excellent luminescent and superior electrical transport characteristics. The polyfunctional ligand dimercaptosuccinic acid (DSA), which has strong binding affinity with AIGS QDs, was employed to reshape the QD surface via passivating the uncoordinated Ga^3+^ and suppressing S vacancies. After DSA passivation, both DAP recombination and non-radiative recombination pathways were effectively decreased, leading to a notable narrowing FWHM to 31 nm and an enhancement of PLQY up to 89%. In addition, the DSA passivation improves the electrical transport properties of QDs, thereby facilitating more efficient charge injection and transport. As a result of the DSA ligand-reshaped treatment, the optimized QLED based on AIGS QDs achieves a high EQE of 8.4% with a narrow FWHM of 31 nm, which represents the highest device performance reported for the AIGS-based QLEDs to date. This work demonstrates the considerable potential of the AIGS QLEDs in the future high-quality lightings and high-definition displays.

## Experimental Section

### Starting Materials

Indium chloride (InCl_3_, 99.9%), gallium chloride (GaCl_3_,99.999%), silver acetate (Ag(OAc), 99.5%), gallium acetylacetonate (Ga(acac)_3_, 99.99%), 1,3-dimethylthiourea (DMTU, 97%), diethylammonium diethyldithiocarbamate (DEA-DDTC, ≥ 97%), tetrahydrofuran (99.5%), n-hexane (≥ 99%), octane (96%), oleylamine (OAm), dimercaptosuccinic acid (DSA) were purchased from Macklin. Trichloromethane (AR) and toluene (AR) were purchased from Luoyang Chemical Factory. Ethyl acetate (AR), isopropyl alcohol (AR) and ethanol (AR) were purchased from Fuyu Reagent. Methanol anhydrous (AR) and acetone were purchased from Fuchen Chemical Reagent. Poly (bis(4-phenyl)(2,4,6-trimethylphenyl) amine) (PTAA), 2,4,6-tris(3′-(pyridin-3-yl) biphenyl-3-yl)-1,3,5-triazine (TmPPPyTz), and poly(ethylene dioxythiophene):polystyrene sulfonate (PEDOT:PSS, AI 4083) were purchased from Xi’an Polymer Light Technology Corp. All reagents were used as received without further purification.

### Preparation of Precursors

#### Gallium Diethyldithiocarbamate (Ga(DDTC)_3_) and Indium Diethyldithiocarbamate (In(DDTC)_3_)

Ga(DDTC)_3_ was prepared following the previous report with some modifications [17], where the volume was expanded and the purification process was optimized. In a nitrogen glovebox, 12 mmol GaCl_3_ (1.056 g) was dissolved in dehydrated toluene (200 mL), followed by the addition of 12 mmol DEA-DDTC (8.006 g) under vigorous stirring. The solution was stirred for another 3 h and taken out of the glovebox. Toluene and most of the diethylammonium chloride, which is the byproduct, were removed by a rotary evaporator. The powder was dissolved in the least amount of chloroform and centrifuged two times (10,000 rpm, 1 min) to achieve purity. The product was recovered by vacuum drying in the 60 °C overnight. To synthesize In(DDTC)_3_, 12 mmol InCl_3_ (2.658 g) and tetrahydrofuran were used instead of GaCl_3_ and toluene, respectively.

#### 0.2 M OAm Solution of GaCl_3_

In a nitrogen glovebox, GaCl_3_ (4 mmol, 704 mg) was dissolved in OAm (20 mL), and heated at 100 °C and stirred into a transparent solution. Before each use, preheat the solution.

#### 0.25 M OAm Solution of Ag(OAc)

Ag(OAc) (5 mmol, 834 mg) was dissolved in OAm (20 mL), and heated at 100 °C and stirred into a transparent solution. After cooling to room temperature, use an organic filter to filter the solution. Before each use, preheat the solution.

### Synthesis of AIGS QDs

#### Synthesis of AIGS with Ga-S Enrichment QDs

The AIGS QDs were synthesized via previous reported two-step method with some modifications [17].

Step Ⅰ: InCl_3_ (0.4 mmol, 88.4 mg), In(DDTC)_3_ (0.1 mmol, 56 mg), and Ga(DDTC)_3_ (0.6 mmol, 308 mg) were mixed with OAm (20 mL) in a 100 mL three-necked flask and heated at 100 °C under vacuum for 20 min. After the powders were completely dissolved, the flask was filled with N_2_, and the heating packet was set at 200 °C. When the temperature of the solution reached 130 °C, 2 mL Ag(OAc) solution was swiftly injected into the flask. After heating at 200 °C for 30 min, the solution was cooled to room temperature using an ice bath. Then, the crude solution, as the AgIn_x_Ga_1−x_S_2_ core solution, was retained for subsequent use.

Step Ⅱ: Ga(acac)_3_ (0.3 mmol, 110 mg), Ga(DDTC)_3_ (0.1 mmol, 52 mg), and DMTU (0.3 mmol, 20 mg) were dissolved in 20 mL OAm, to which 3 mL AgIn_x_Ga_1−x_S_2_ core solution was introduced. The solution was evacuated at 100 °C for 20 min and switched to an N_2_ atmosphere. The temperature was rapidly increased to 230 °C and afterward to 280 °C at a rate of 2 °C min^−1^. When the core/shell QD solution maintained for 30 min at 280 °C, 3 mL GaCl_3_/OAm solution was injected and maintained for further 30 min, then cooled to room temperature using an ice bath.

Purification: The AIGS/GS crude solution was precipitated via methanol through centrifugation for the first time, then the precipitate was further purified via toluene/ethanol mixture for the second time, the final product was dispersed in octane for further use.

#### Synthesis of LR AIGS QDs

The synthesis process is identical to that of control AIGS QDs, except that during the first and second purification processes, DSA was incorporated into the AIGS/GS crude/dispersed solution for surface reshaping. Typically, for purification of the LR-treated QDs, 10 mg DSA was added into the as-synthesized AIGS/GS crude solution, and stirred at room temperature under N_2_ flow for 5 min. Then, the precipitate was collected through the purification of ethanol. The first-purified QDs were dispersed in toluene; another 10 mg DSA was added into the QD/toluene solution at atmosphere (stirred for 5 min) for further ligand treatment. Subsequently, the LR AIGS QDs were collected through the purification of ethanol again. Other reaction conditions were consistent.

### Fabrication of AIGS QLEDs

Device construction: The patterned ITO was treated with deionized water, isopropyl alcohol, and acetone by ultrasonic treatment for 15 min, and then treated with ultraviolet-ozone for 20 min before use. PEDOT: PSS was filtered by 0.22 µm filter, and then spin coated on the ITO glass substrate at 4000 rpm for 60 s and heat at 140 °C for 15 min. Then, PTAA (6 mg mL^−1^ in chlorobenzene) and AIGS/GS QDs were deposited layer by layer through spin coating at 3000 rpm for 60 s (PTAA layer was heat at 120 °C for 20 min). Finally, 3P(TmPPPyTz) (40 nm)) and LiF/Al electrodes (1 nm/100 nm) were deposited using a thermal evaporation system under a vacuum of ~ 5 × 10^–4^ Pa. The light-emitting area of the device was 2.25 mm^2^ as defined by the overlapping area of ITO and Al electrodes.

### Characterization and Measurement

The steady-state photoluminescence spectra were tested through a spectrofluorometer (Hitachi Fluorescence Spectrophotometer F-4700). The ultraviolet–visible (UV–vis) absorption spectra were measured using a UV–vis spectrophotometer (Hitachi UV–Visible/NIR Spectrophotometer UH5700). The QD morphologies were observed using a transmission electron microscopy (TEM) instrument (Hitachi, H-7650) at an acceleration voltage of 100 kV, whereas high-resolution TEM (HRTEM) images were obtained using a 200 kV TEM (JEOL, JEM-2100). Powder X-ray diffraction (XRD) analysis was performed using an X-ray diffractometer (Rigaku, SmartLab) equipped with a parallel beam/parallel slit analyzer. The chemical composition of the QDs was determined using an inductively coupled plasma atomic emission spectrometer (Shimadzu, ICPS-7510). X-ray photoelectron spectroscopy (XPS) measurements were employed using a VG ESCALAB 220i-XL spectrometer with a 300 W Al Kα radiation source. Fourier transform infrared spectroscopy (FTIR) was detected using the infrared spectrometer (Nicolet IS10, USA). Variable temperature PL spectra are measured using a FLS1000 photoluminescence spectrometer (Cryo77, Tianjin Orient-KOJI Instrument and TAP-02, Tianjin Orient-KOJI Instrument). Variable power PL spectra were measured by a laser diode controller (ADR-1805). The PLQY was measured using a Horiba Fluorolog system equipped with a single grating and a Quanta-Phil integration sphere coupled to the Fluorolog system. Time-resolved PL (TRPL) decay curves of the films were obtained using an FLS-1000 spectrofluorometer. The ultrafast TA measurements were performed on a femtosecond (fs) pump–probe system (Helios, Ultrafast System LLC) under ambient conditions. The atomic force microscopy (AFM) and conductive AFM images and roughness analysis were obtained using the AFM system (INNOVA AFM, Bruker). The EL spectra, *L–J–V* characteristics, EQE, and operational lifetime of the device were collected under atmosphere without encapsulation using a LED testing system which was conducted by a Keithley 2602B source meter and a Minolta CS-2000 system.

## Results and Discussion

### Optimized AIGS QD Surface by Ligand-Reshaped Strategy

**Fig. 1:**
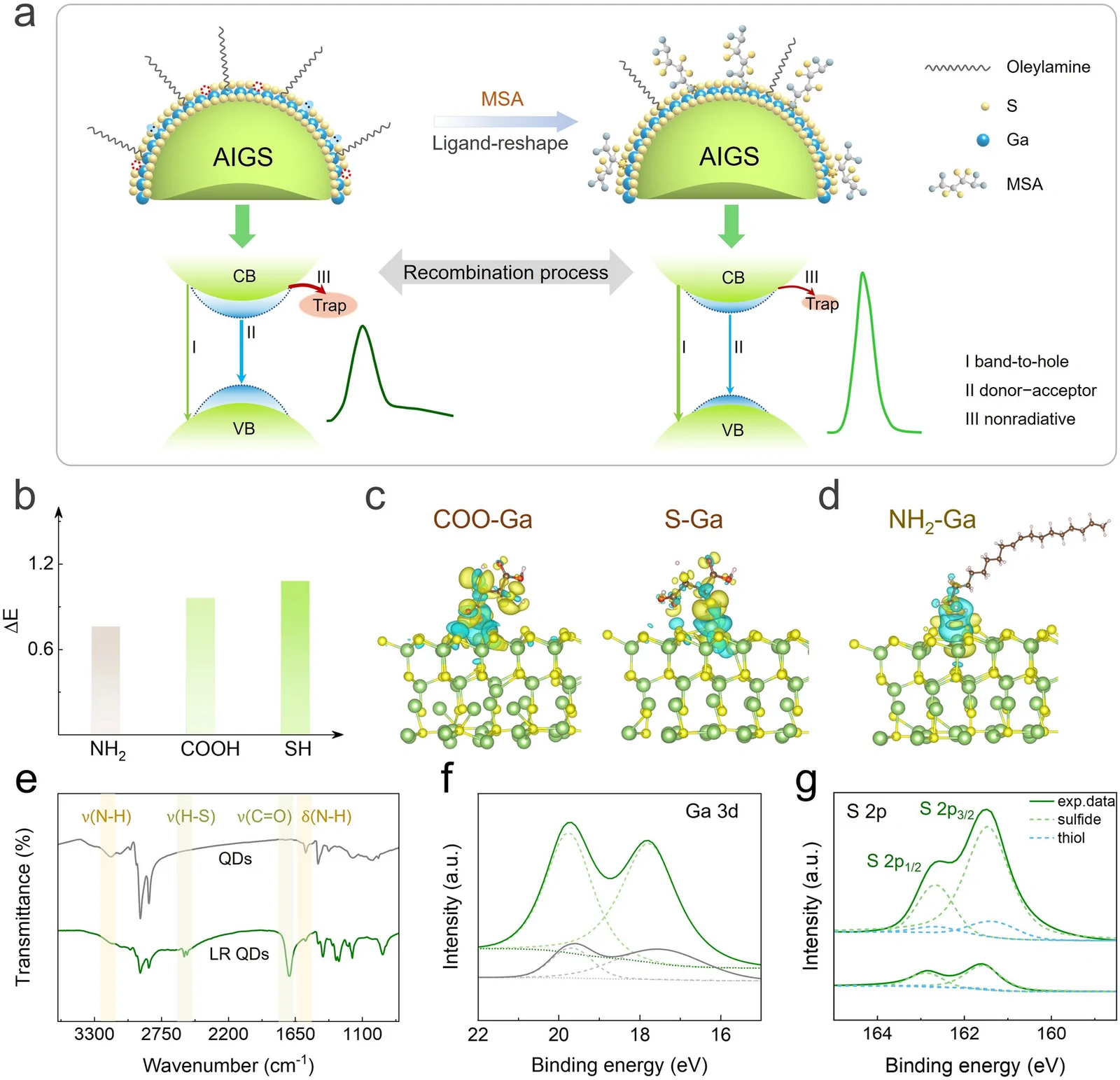
**a** Schematic description of recombination process before and after resurfacing treatment. **b** DFT calculated adsorption energy of amino from oleylamine and carboxyl and sulfydryl from DSA with Ga on GS surface. The difference charge-density map of **c** DSA and **d** oleylamine adsorbing on the GS surface. **e** FTIR spectra of control and LR QDs. High-resolution XPS spectra of the control and LR QDs: **f** Ga 3*d*, and **g** S 2*p*

AIGS QDs with a thin Ga-S enrichment coating (~ 1 nm) were synthesized via a classical two-step process [17]. Although the Ga-S surface coating effectively narrows the PL spectrum from 150 to 37 nm (Fig. S1), the coating is generally very thin (~ 1 nm). Consequently, the surface state is associated with various defects, e.g., the absent S and uncoordinated Ga^3+^. The V_S_ and uncoordinated Ga^3+^ would be the DAP recombination channel and non-radiative recombination center [17, 18], leading to broadened FWHM and reduced PLQY (Fig. 1a). Previous reports [18–20] have demonstrated that both band-to-hole and DAP recombination serve as the dominant contributors to the PL emission of AIGS/GS QDs. Whereas DAP recombination, which is closely related to inherent defects (e.g., V_S_), primarily accounts for the broadened PL, while defect-related non-radiative recombination, originating from surface traps, leads to low PLQY [10, 18, 21, 22]. Therefore, reducing V_S_ or/and uncoordinated Ga^3+^ trap state is expected to suppress DAP and non-radiative recombination pathways, thereby narrowing FWHM and improving the PLQY [10, 18].

Thus, we propose a ligand-reshaped (LR) strategy via introducing the polyfunctional ligand DSA, which can strongly bind to surface Ga^3+^ through carboxyl and sulfydryl groups (Figs. 1a and S2), as well as filling V_S_ through S from sulfydryl, to passivate the QDs and repair the surface states. In order to support this design, the formation energy of surface Ga vacancy (V_Ga_) and S vacancy (V_S_) on Ga-S model was calculated through the density functional theory (DFT) (Fig. S3). The lower defect formation of V_S_ indicates that V_S_ is more prone to form on GS surface, leaving uncoordinated Ga^3+^, which would broaden the FWHM and degrade the PL performance due to DAP and non-radiative recombination triggered by these defects. To mitigate these detrimental defects, we put forward the DSA LR passivation approach to optimize the surface state of AIGS QDs.

Compared with oleylamine-passivated QDs (Fig. 1b–d), both the carboxyl (0.96 eV) and sulfydryl (1.08 eV) of DSA exhibit strong interaction with surface Ga^3+^, showing stronger adsorption energy with AIGS QDs than that of control QDs (0.76 eV). The interaction was further confirmed through FTIR and XPS. In the LR DSA-passivated QDs (LR QDs), the stretched vibration peaks of H–S (2550 cm^−1^) and C=O (1690 cm^−1^) from DSA are clearly observed (Fig. 1e), while the stretched vibration peak (3150 cm^−1^) and bending vibration peak (1550 cm^−1^) of N–H from oleylamine are significantly weakened, indicating the effective displacement of oleylamine by DSA and firm binding of DSA to QD surface for efficient defect passivation. The high-resolution XPS spectra presented in Fig. 1f, g further confirm the changes in the surface state. The lower-intensity peak from thiol is hardly observed in the control QDs, while the increased peaks ascribed to thiol in the LR QDs prove the successful introduction of DSA. In addition, for the LR QDs, the binding energy of Ga 3d shifts to higher energy due to the interaction surface Ga^3+^ with thiol/carboxyl group from DSA, while the splitting peaks of S 2*p* at 162.6 and 161.6 eV (correspond to the S 2*p*_1/2_ and 2*p*_3/2_) shift to lower binding energy attributed to the formation of Ga-S bonds after the introduction of DSA. ^1^H and ^13^C NMR signal (Fig. S4) from carboxyl of DSA shift downfield after being introduced into AIGS QDs, demonstrating the bonding between carboxyl with Ga^3+^. The decreased electron paramagnetic resonance (EPR) signal (*g* = 2.005) in LR QDs (Fig. S5) exhibited the decrease V_S_ after LR treatment [23].

**Fig. 2:**
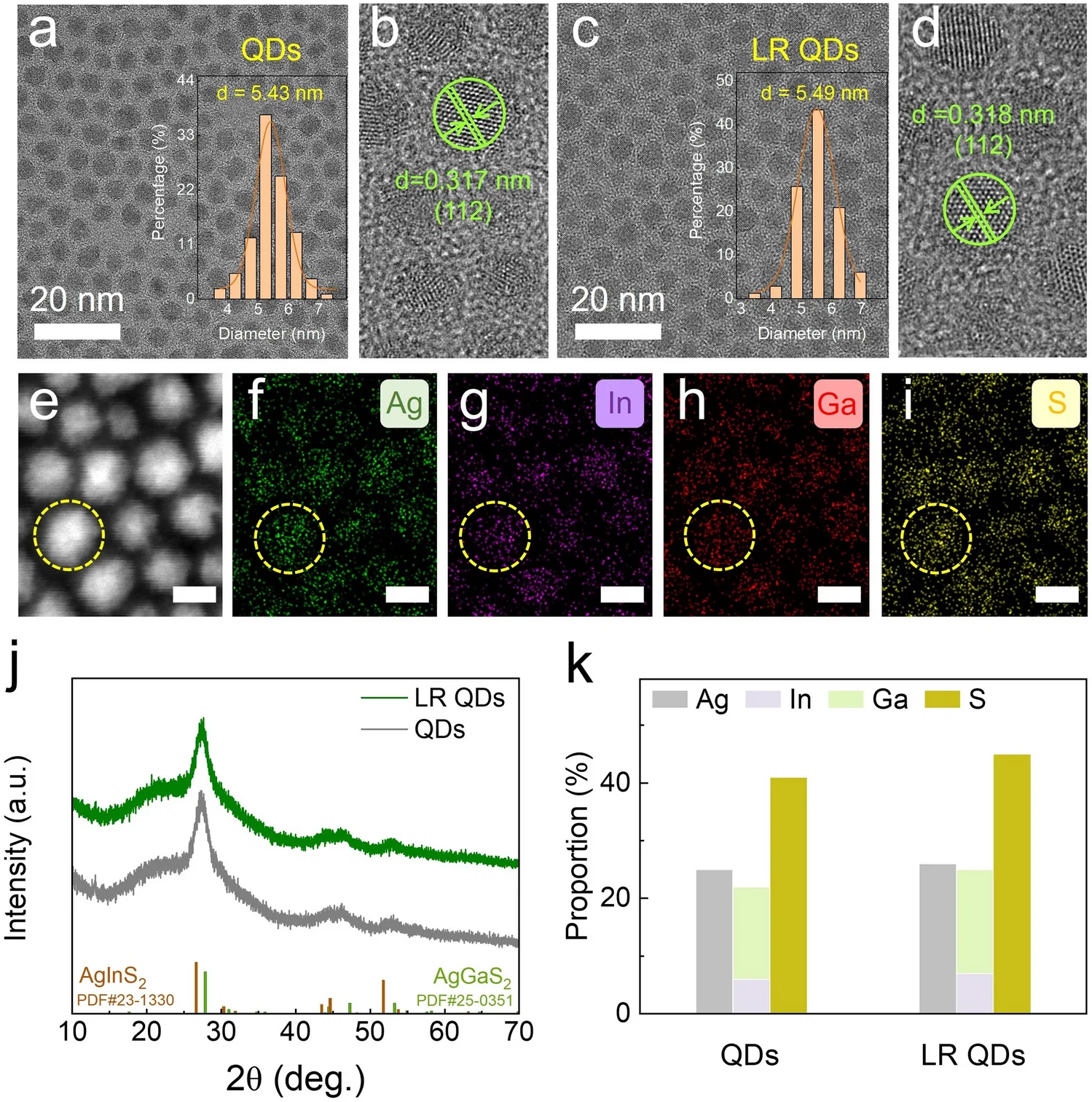
**a** TEM and **b** HRTEM images of control QDs. **c** TEM and **d** HRTEM images of LR QDs. **e–i** Energy-dispersive spectroscopy elemental maps of Ag/In/Ga/S of LR QDs, the scale bar is 5 nm. **j** XRD patterns and **k** ICP-MS characterization of control and LR QDs

The microstructures of AIGS QDs before and after LR treatment are shown in Fig. 2. The LR DSA-passivated QDs exhibit similar microstructure compared with control QDs, with both displaying a spherical morphology with an average size of 5.4 nm (Fig. 2a, c). While LR QDs exhibit a narrower size distribution. In comparison, the AIGS core without Ga-S enrichment has an average size of 4.18 nm (Fig. S6), indicating that the Ga-S coating is thin (~ 1 nm), consistent with previous report [17, 24]. HRTEM images reveal interplanar spacing of 3.17 Å (control QDs) and 3.18 Å (LR QDs), corresponding to the (112) lattice of tetragonal AIGS (Fig. 2b, d). Furthermore, energy-dispersive X-ray spectroscopy (EDS) elemental mappings (Figs. 2e-i and S7) illustrate the uniform distribution of Ag, In, Ga, and S across the QDs. XRD patterns presented in Fig. 2j reveal that both control and LR QDs exhibit the diffraction peak characteristics of AgInS_2_ (PDF#23–1330) and AgGaS_2_ (PDF#25–0351), corresponding to previous reports [7, 24, 25]. Above results demonstrate that the introduction of DSA primarily affects the QD surface without obviously altering the morphology and crystal structure of AIGS QDs. Furthermore, the increased proportion of S, as revealed by inductively coupled plasma (ICP) (Fig. 2k and Table S1), is attributed to the supplement of S-containing DSA, which indicates that DSA located on QD surface can reshape the surface state to some extent.

### Optical Properties and Carrier Recombination Characteristics of AIGS QDs

**Fig. 3:**
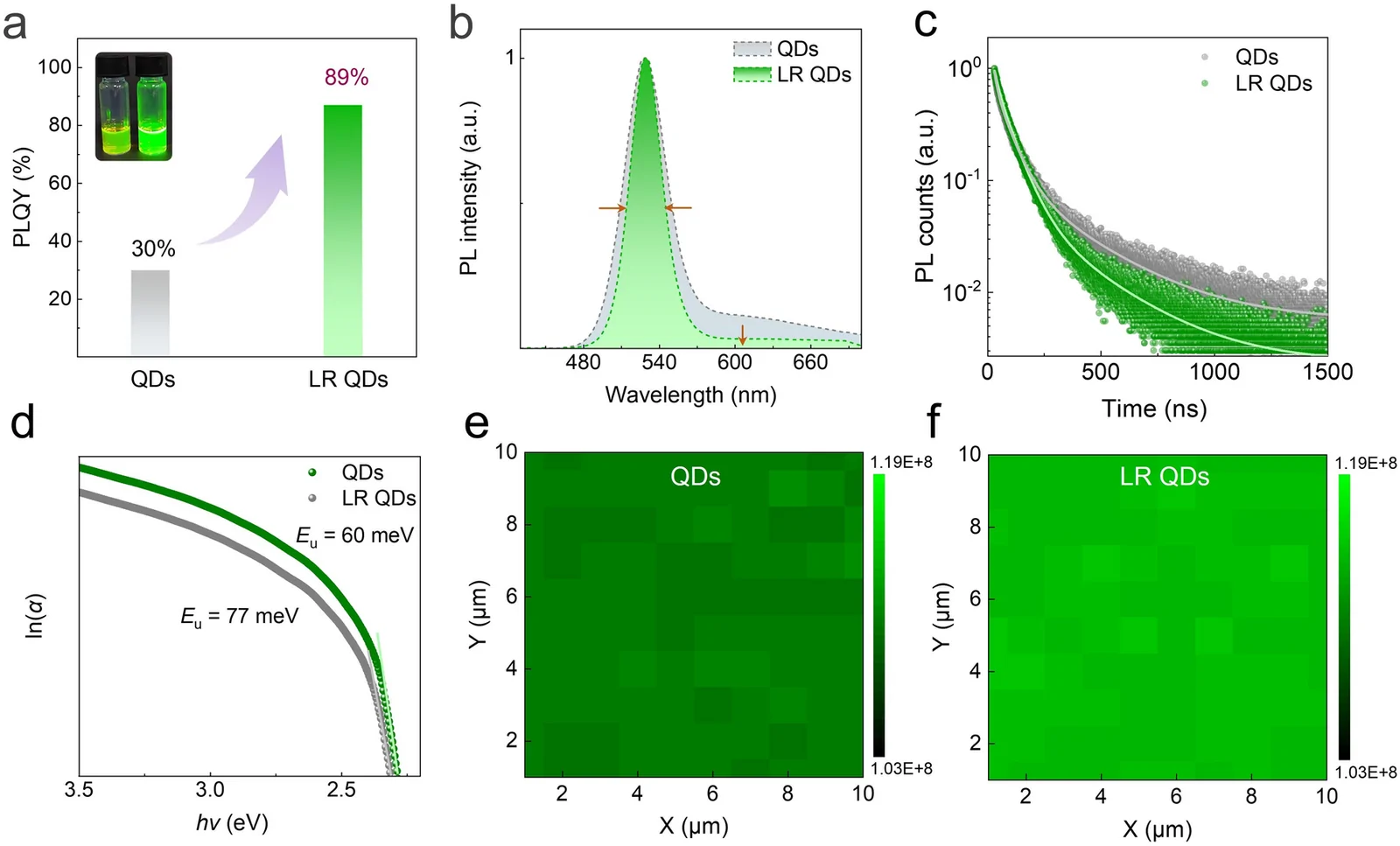
**a** PLQY of control and LR QDs, inset is the photograph of colloidal QDs under UV lamp. **b** PL spectra and **c** time-resolved PL spectra of control and LR QDs. **d** Relationship of ln(*α*) versus *hν* for the calculation of Urbach energy. PL mapping of **e** control and **f** LR QDs with an area of 10 μm × 10 μm

Benefiting from the LR process, the surface state of QDs is effectively passivated, resulting in brighter emission for the DSA-passivated QDs (right sample in the inset photograph in Fig. 3a). This enhancement is further evidenced by the PLQY results, which show that the DSA-passivated QDs exhibit a PLQY of 89%, significantly higher than that of control QDs (30%) (Figs. 3a and S8). In addition, the DSA-passivated QDs display a narrower FWHM at main peak from 37 to 31 nm due to the narrower size distribution and decreased surface defects, as well as obviously suppressed broad PL tailing (600 nm) related to DAP recombination (Fig. 3b). Furthermore, the time-resolved photoluminescence (TRPL) was recorded to analyze the carrier recombination dynamics in QDs, as shown in (Fig. 3c). The PL decay curve can be well fitted by the triexponential decay function:

1 $$I(t) = A_{1} e^{{ - t/\tau_{1} }} + A_{2} e^{{ - t/\tau_{2} }} + A_{3} e^{{ - t/\tau_{3} }}$$

*A*_1_, *A*_2_, and *A*_3_ represent the amplitude of the decay components at *t* = 0. The fast decay component (*τ*_1_) is indicative of non-radiative recombination caused by surface defect states. The second (*τ*_*2*_) and third components (*τ*_*3*_) represent band-to-hole recombination and DAP recombination, respectively [17, 24, 26].

2 $$\tau_{{{\mathrm{avg}}}} = \frac{{A_{1} \tau_{1}^{2} + A_{2} \tau_{2}^{2} + A_{3} \tau_{3}^{2} }}{{A_{1} \tau_{1} + A_{2} \tau_{2} + A_{3} \tau_{3} }}$$

The corresponding fitting results are summarized in Table S2. In the time-resolved PL decay analysis, these three components exhibit a competitive relationship. Benefiting from surface state reshaping, the ratios of DAP recombination and defect-related non-radiative recombination are reduced, while the band-to-hole recombination is remarkably increased, which are the main reasons for the narrowed FWHM and improved PLQY. The Urbach energies (*E*u) of the QDs were calculated to further prove the reduction in non-radiative recombination associated with defects (Fig. 3d). Generally, the *E*u reflects the tail of localized states within the bandgap of QDs, which is positively correlated with the density of localized states near the band edges [25, 27]. The results show that the *E*u of LR QDs is 60 meV, lower than that of control QDs (77 meV), further indicating the effective suppress of non-radiative recombination. Contributing to the reduction in defects and improved radiative recombination, the LR DSA-passivated QD film exhibits enhanced PL intensity and uniformity, as illustrated by the micro-PL mapping (Fig. 3e, f). Moreover, the LR QD film exhibits a lower surface roughness and a more uniform morphology (Fig. S9), which constitutes one of the important prerequisites for the high-efficiency QLEDs.

**Fig. 4:**
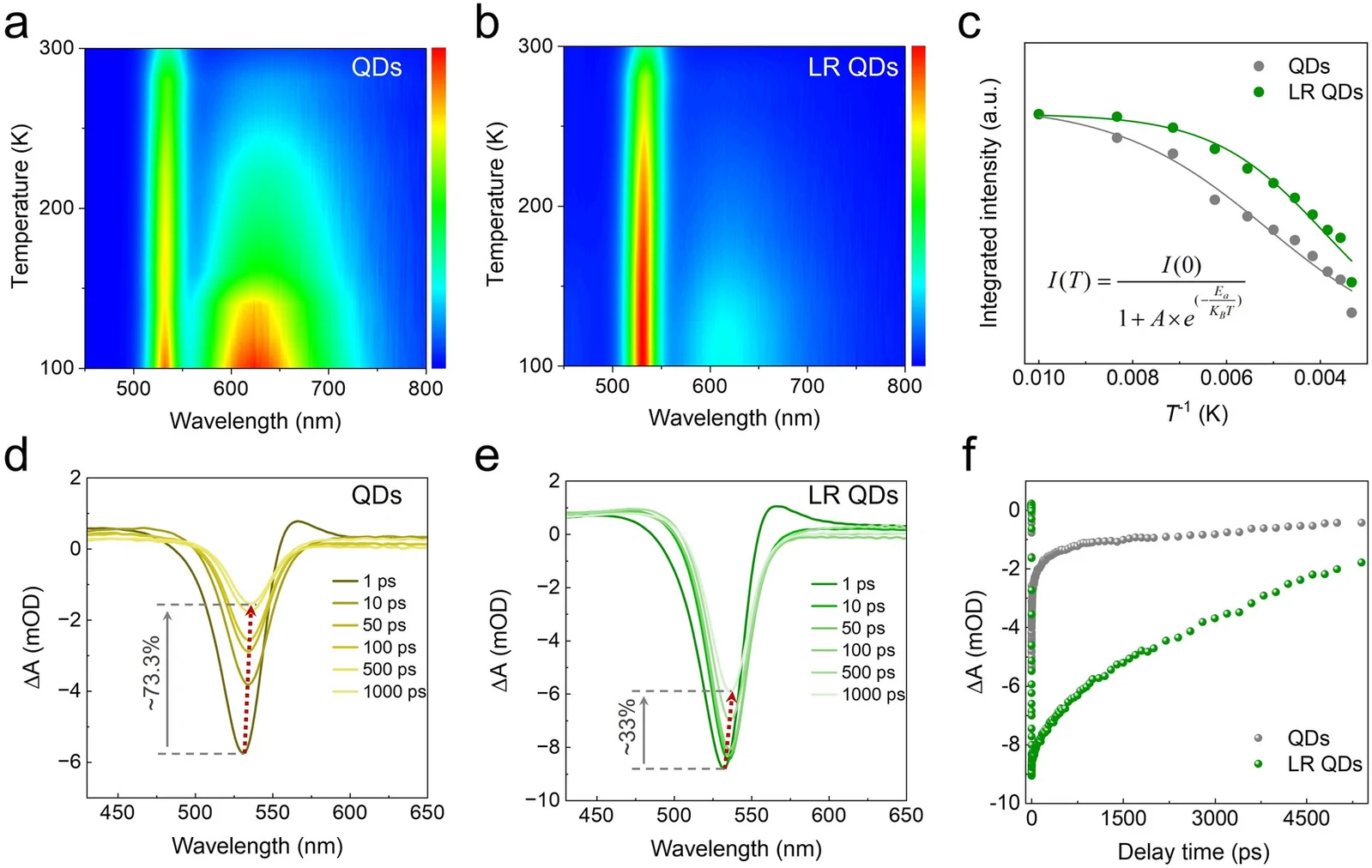
**a** Temperature-dependent PL spectra of control and LR QDs. The color bar represents the normalized PL intensity. **b** Plot of integrated PL intensity fitted with the Arrhenius equation. **c** fs-TA spectra of **d** control and **e** LR QDs. **f** Kinetic traces of ground-state bleaching (GSB) maximum of control and LR QD

The recombination characteristics of the QDs were further analyzed through temperature-dependent PL spectra and femtosecond transient absorption (TA) spectroscopy. The temperature-dependent PL spectra were recorded from 100 to 300 K under 365 nm excitation, as presented in Fig. 4a, b. It is evident that the second emission peak related to DAP recombination becomes more pronounced with decreasing temperature, whereas DAP recombination in LR DSA-passivated QDs is significantly suppressed. Meanwhile, the LR QDs show a lower degree of PL intensity degradation with the increasing temperature compared to the control QDs. To gain deeper insight into the carrier recombination mechanisms within the QDs, the integrated PL intensity was plotted as a function of the inverse temperature and fitted it with the Arrhenius equation [28–30] (Fig. 4c):

3 $$I\left( T \right) = \frac{{I_{0} }}{{1 + Ae^{{\frac{{ - E_{a} }}{{k_{B} T}}}} }}$$

*I(T)* is the integrated PL intensity, *I*_*0*_ is the 0 K integrated intensity, *E*_*a*_ is the activation energy of non-radiative recombination centers, and *k*_*B*_ is the Boltzmann’s constant. The calculated *A* value [28] proportional to the non-radiative carrier density decreases from 15.7 for control QDs to 10.9 for LR QDs, indicating an improved radiative recombination efficiency for LR QDs.

Moreover, femtosecond TA spectroscopy was employed to analyze the exciton state relaxation dynamics in QDs (Fig. S10). The TA spectra reveal that both samples exhibit a pronounced photobleaching peak at ≈ 505 nm upon excitation at 365 nm (Fig. 4d–f), which is attributed to ground-state bleaching. Over time, the ΔA is decreased by only 33% for LR QDs, whereas the untreated QDs shows a decrease of 73.3%. The optical density (ΔA) is proportional to the exciton density in the lowest excited state [16, 31]. Additionally, the smaller redshift of the bleaching peak in GS QDs also confirm the reduction of sub-bandgap defects [32]. Concurrently, the carrier dynamics were analyzed through kinetic fitting of ground-state bleach (GSB) as shown in Fig. 4f. The triexponential fitting recovery features are detailed in Table S3. The first component, associated with defects near conduction band, and the third component, related to DAP recombination, are significantly suppressed, while the second component corresponding to band-to-hole recombination is competitively enhanced, which is agreement with the fitting results of TRPL. Attributing to the effective defect passivation, the PL stability of QDs has also been improved that is verified through the PL attenuation test under atmosphere storage, UV irradiation and heating at 80 °C (Fig. S11).

### Electrical Properties and Defect State Analysis of AIGS QDs

**Fig. 5:**
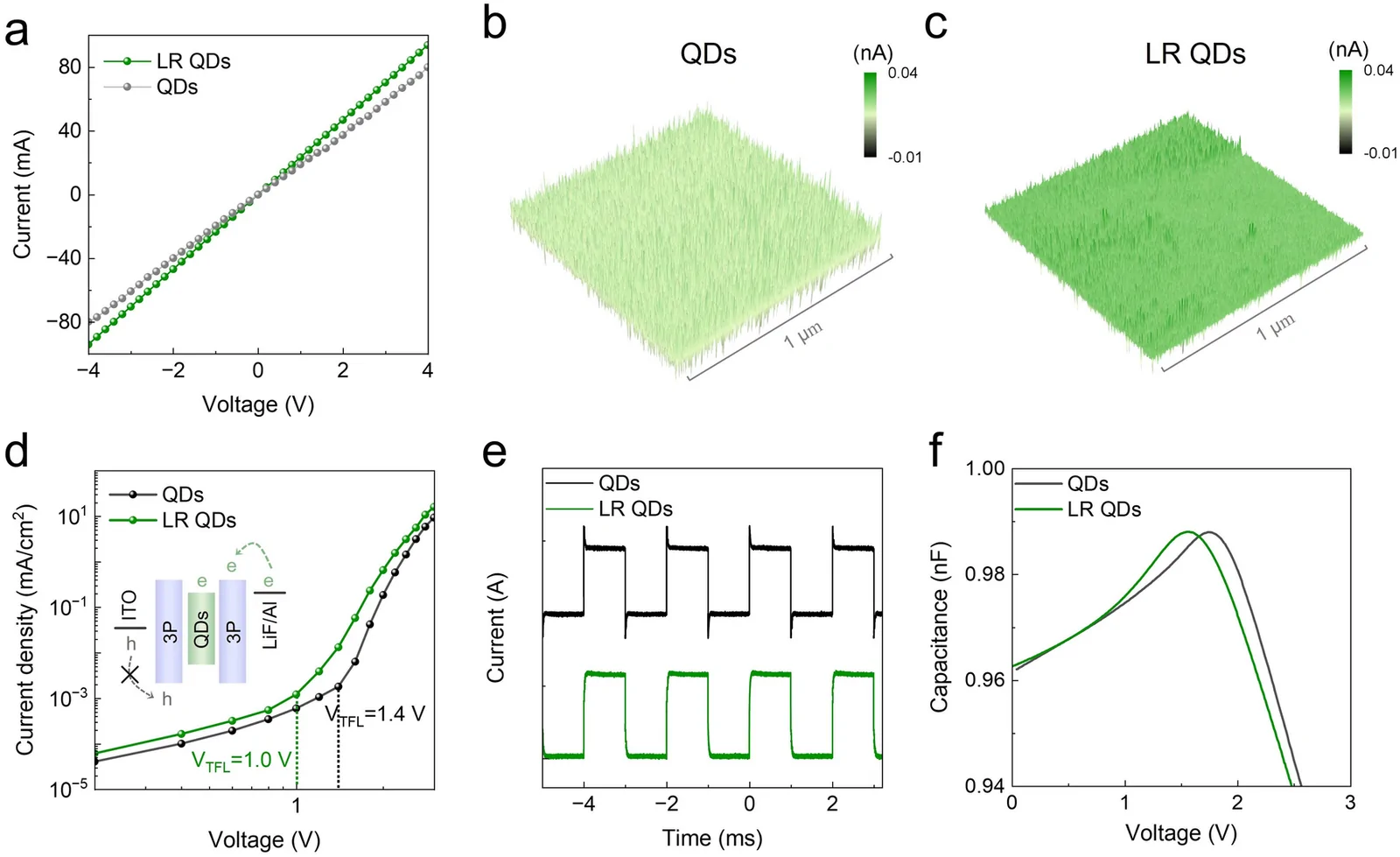
**a** *I*-*V* curves of devices with a structure of ITO/QDs/Au. C-AFM of **b** control QD film and **c** LR QD film. **d** *J-V* curves of the electron-only device. **e** *I-t* curves and **f** *C-V* curves of control and LR QLEDs

In addition, benefiting from the effective ligand replacement of oleylamine with short-chain DSA, the LR QD film exhibits enhanced electrical conductivity. The *I*-*V* curves (Fig. 5a) reflect the electrical conductivity of QDs through *σ* = *dI*/*SV* [33], it can be seen that LR QDs exhibit better electrical conductivity. Furthermore, conductive atomic force microscopy (CAFM) reveals that the current of QDs increases after LR treatment, verifying the increase in the conductivity of LR QDs (Fig. 5b, c). The improved conductivity facilitates more efficient injection and transport of charge carriers.

The carrier behaviors under electric field are further discussed below. The defects are further quantitatively analyzed by space-charge-limited current (SCLC) by the “electron-only” device with a structure of glass/indium tin oxide ITO/3P/QDs or QDs + DSA/3P/Au, as shown in Fig. 5d. The sharp rise of the current density–voltage (*J-V*) curve correlates with a trap-filled limit (TFL), where the defects are occupied by charge carriers. The defect density is calculated according to Eq. 4 [1, 34]:

4 $$N_{defects} = \, \left( {2\varepsilon \varepsilon_{0} V_{TFL} } \right)/(eL^{2} )$$

where *ε* represents the dielectric constants of the AIGS (Fig. S12), *ε*_*0*_ represents the dielectric constant of vacuum permittivity, *L* is the thickness of AIGS QDs film, and *e* is the elementary charge. The electron trap density of LR QDs shows a lower trap-state density (1.33 × 10^19^ cm^−3^) compared to that of control QDs (1.86 × 10^19^ cm^−3^). Meanwhile, the transient photocurrent excited by a 405 nm laser exhibits that the control QDs display slower turn-on and turn-off dynamics, which can be attributed to the time taken for the defect trapping/detrapping or slower DAP capture processes to reach steady state following light switching (turn-on/off) (Fig. 5e). Furthermore, the capacitance–voltage (*C-V*) curves were conducted on QLEDs before and after LR DSA-treatment as shown in Fig. 5f. A sharp increase in capacitance at low voltage indicates the accumulation of injected major charge carriers, while the recombination of accumulated major charge carriers with injected minor carriers at high voltage leads to the decreasing capacitance [35]. The LR QLEDs exhibit peak capacitance at lower voltage compared to the control device, suggesting the faster charge transport and more efficient electron–hole recombination in LR QLEDs.

### Construction and Performance Characterization of QLEDs

**Fig. 6:**
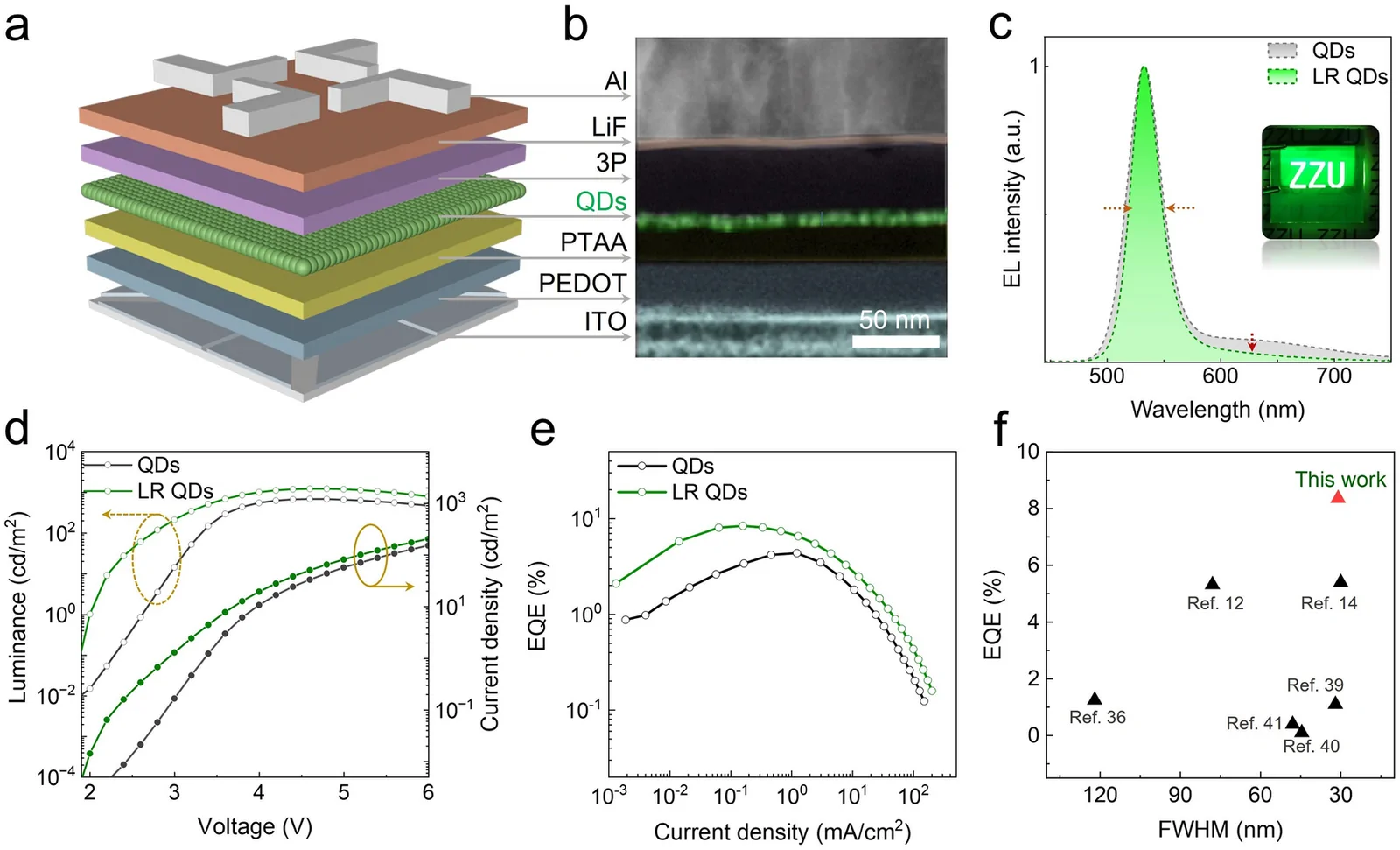
**a** Structural schematic diagram and **b** cross-sectional STEM image of the QLED device. **c** EL spectra, **d** *L-J-V* curves, and **e** *EQE-J* curves of control and LR QLEDs. **f** Summarized EQE and FWHM of reported AIGS QLEDs

Inspired by the improved optical and electrical properties of LR DSA-passivated AIGS QDs, the QLEDs with the typical device architecture-ITO/PEDOT/PTAA/QDs/3P/LiF/Al (Fig. 6a) is constructed. The corresponding cross-sectional scanning transmission electron microscopy (STEM) image is presented in Fig. 6b. The resulting QLED based on LR DSA-passivated QDs shows a bright green emission (inset of Fig. 6c), with the electroluminescence (EL) peak at 531 nm (Fig. 6c). Compared to the control QDs, the DSA-passivated QD-based device maintains the same EL peak position, while exhibiting a narrower FWHM (from 37 to 31 nm) and suppressed DAP-related asymmetric tailing, which is consistent with the result of PL. Moreover, the voltage-dependent EL spectra (Fig. S13) reveal that the DAP-related asymmetric tailing becomes more pronounced as the voltage increases in the control device. After LR DSA treatment, the asymmetric tailing is significantly suppressed and exhibits minimal variation across increasing voltage, indicating the efficient surface defect passivation. And the stability of the EL spectra has also been significantly improved, as the CIE chromaticity coordinates shown in Fig. S14.

The representative luminance-current density–voltage (*L-J-V*) plots of the QLEDs before and after LR DSA treatment are shown in Fig. 6d, the LR device exhibits increased current density due to the improved electrical conductivity. As a result of the enhanced radiative recombination and efficient charge injection, the LR QLEDs demonstrate higher brightness with a maximum value of 1219 cd m^−2^ and lower turn-on voltage of 2.0 V compared to that of control device ((*L*_max_ = 684 cd m^−2^, *V*_turn-on_ = 2.8 V). Accordingly, the LR QLEDs achieve a peak EQE of 8.4% (Fig. 6e), which is about twofold than that of control device (EQE_max_ = 4.3%). The EQE value ranks among the highest reported for the AIGS-based QLEDs (Fig. 6f and Table S4), which presents new opportunities for the heavy-metal-free QLEDs [10, 26, 36–41]. Furthermore, the operational lifetime was tested at initial luminance of 100 cd m^−2^, as shown in Fig. S15, the control device exhibits T50 lifetime of 1 h, while that of DSA-treated device is improved to 5 h.

## Conclusions

In summary, we developed a LR approach using DSA, a short-chain ligand with carboxyl and sulfydryl groups, to modify the surface of AIGS QDs. The multifunctional DSA effectively coordinates with surface defects, enabling simultaneous defect passivation and reconstruction of incomplete surface shells, which can significantly reduce non-radiative recombination and donor–acceptor pair (DAP) recombination in AIGS QDs. Consequently, the PLQY of the LR AIGS QDs was enhanced from 30% to 89%, the FWHM was narrowed from 37 to 31 nm. Additionally, the electrical conductivity of the QD film was substantially improved due to the replacement of long-chain oleymine with DSA. Importantly, the QLED fabricated using the AIGS QDs manifested the high EQE of 8.4% with a narrow FWHM of 31 nm, which represents the highest device performance reported so far for the AIGS QD-based LEDs. We anticipate that this surface engineering strategy could be extended to other I-III-VI QD systems, promoting the application of heavy-metal-free QDs in lighting and display technologies.

## Supplementary Information

Below is the link to the electronic supplementary material.

Supplementary file1 (DOCX 3304 KB)
