# Supplementary material for: Efficient and Pure I-III-VI AIGS Quantum Dot-Based Light-Emitting Diodes via Ligand-Reshaped Surface State
Source: Nanomicro Lett. 2026 Feb 9;18:242. doi: 10.1007/s40820-026-02086-y (PMC12886609; doi:10.1007/s40820-026-02086-y)
Supplement: Supplementary file 1 — Supplementary file1 (DOCX 3304 KB) [file 40820_2026_2086_MOESM1_ESM.docx]

Supporting Information for

**Efficient and Pure I-III-VI AIGS Quantum Dot-based Light-Emitting Diodes via Ligand-Reshaped Surface State**

Leimeng Xu^1^, Jianpeng Zhao^1^, Jindi Wang^1^, Jisong Yao^1^, Shalong Wang^1^, Zhi Wu^1^, Jizhong Song^1^*

^1^Key Laboratory of Materials Physics of Ministry of Education, Laboratory of Zhongyuan Light, School of Physics, Zhengzhou University, Daxue Road 75, Zhengzhou 450051, P. R. China

*Corresponding author. E-mail: [songjizhong@zzu.edu.cn](mailto:songjizhong@zzu.edu.cn) (Jizhong Song)

**S1 Method of density functional theory simulations**

DFT calculations [S1, S2] were performed using the Vienna ab initio simulation package (VASP). The generalized gradient approximation (GGA) in the form of Perdew-Burke-Ernzerhof (PBE) [S3] was adopted as the exchange correlation functional. The energy cutoff of 450 eV and the k-point meshes of 1×1×1 were proposed to carry out geometry optimization and electronic structure calculation. During the geometry optimization, the entire system is considered to have successfully converged until the convergence thresholds of maximum force and energy were smaller than 0.05 eV/A and 1.0×10^-5^ eV/atom, respectively. The vacuum slab was set as 15Å to avoid interactions between neighboring structures.

**S2 Supplementary Figures and Tables**


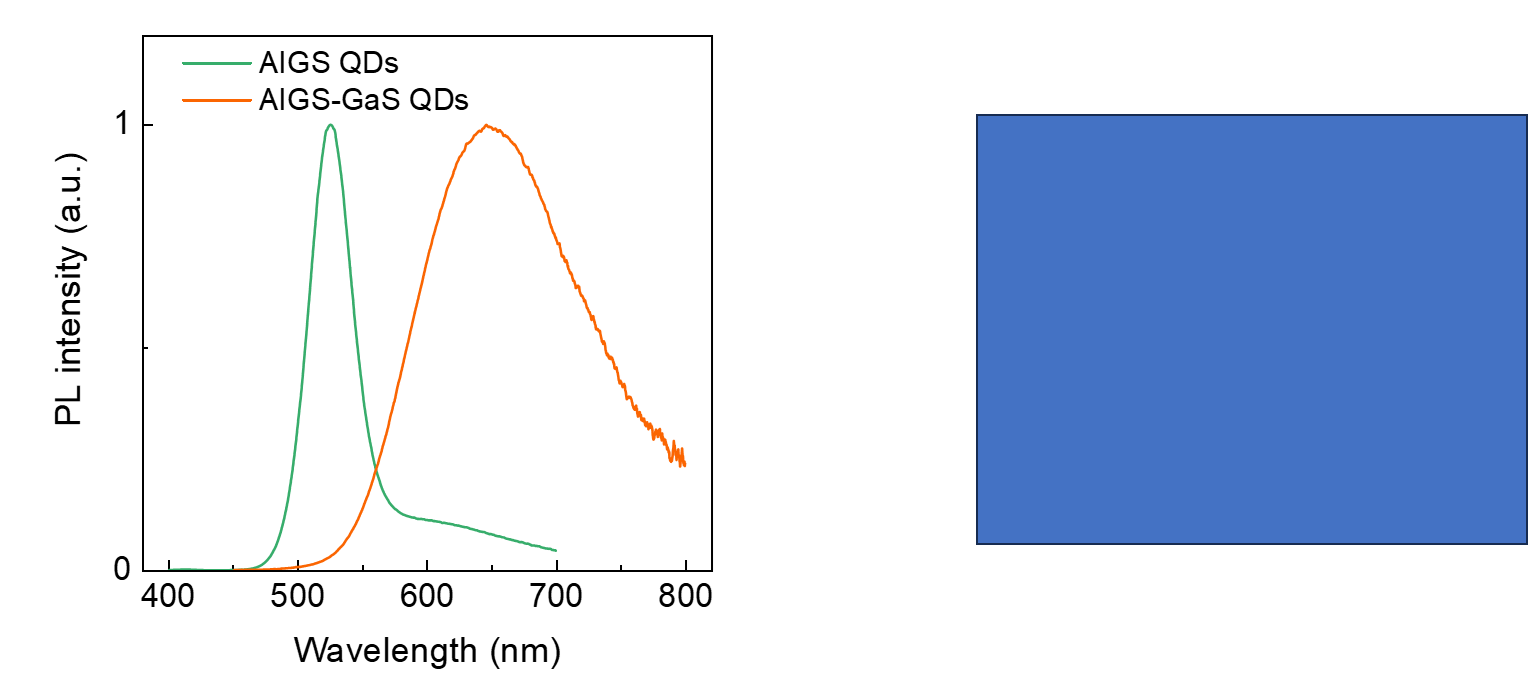


**Fig. S1** PL spectra of AIGS QDs before and after GS coating


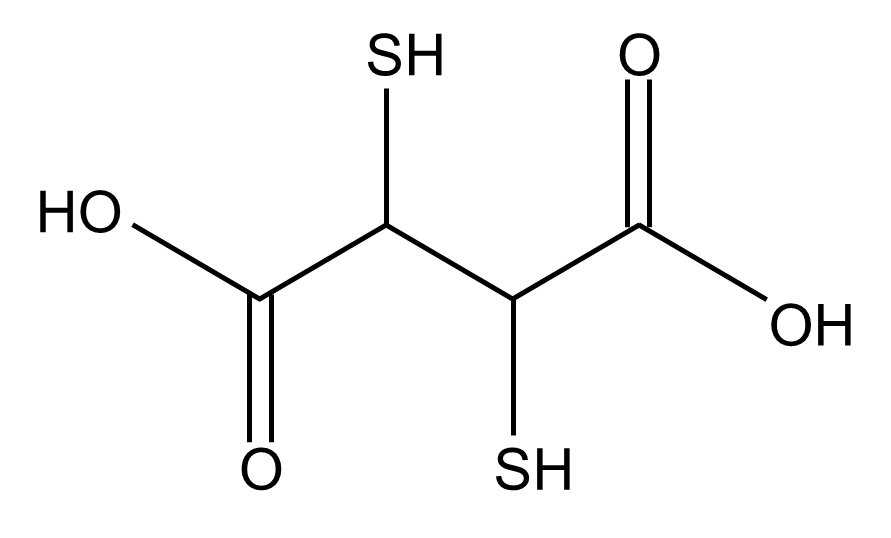


**Fig. S2** The molecular structure of DSA


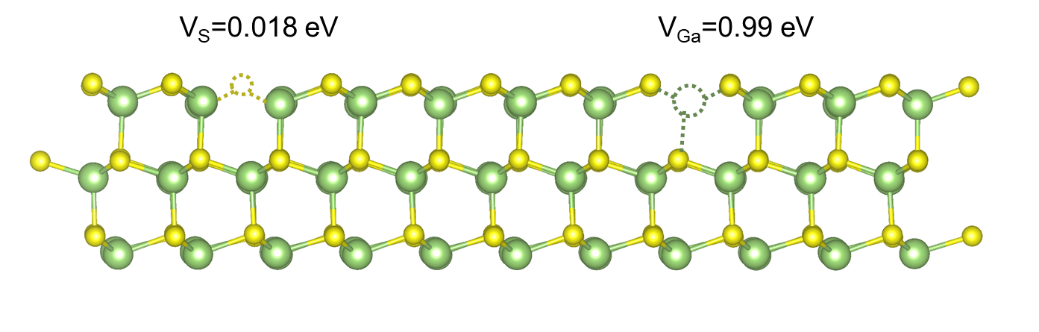


**Fig. S3** Schematic diagram of V_S_ and V_Ga_ on GS surface and their corresponding vacancy formation energy


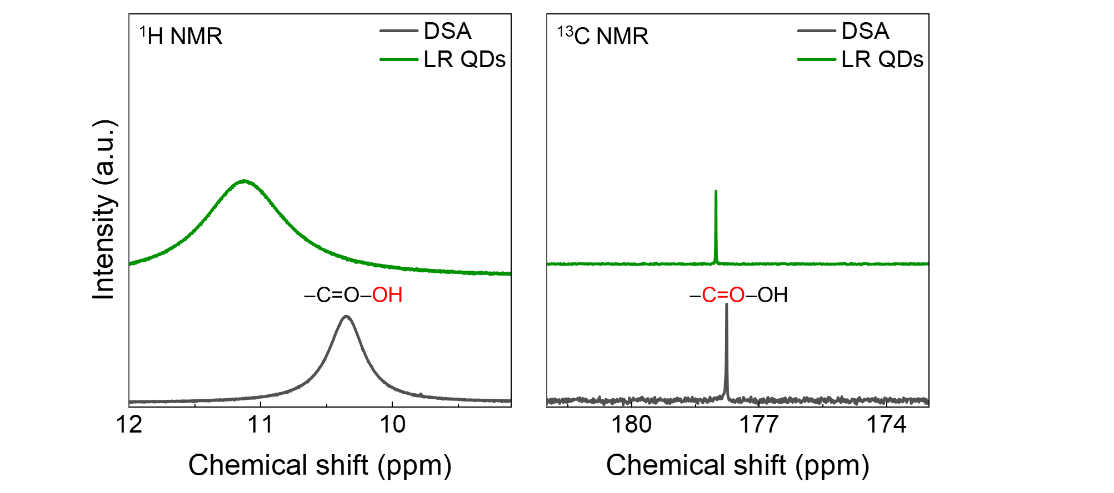


**Fig. S4** ^1^H and ^13^C NMR spectra of DSA and LR QDs


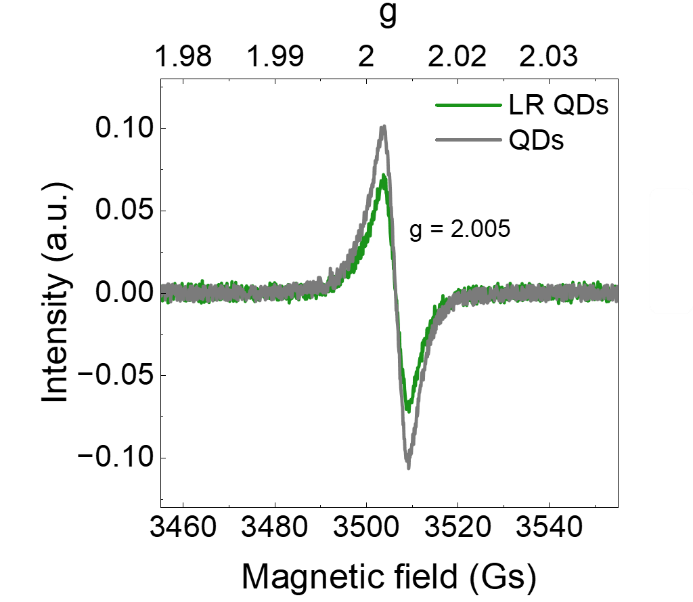


**Fig.** **S5** Electron paramagnetic resonance (EPR) spectra of QDs and LR QDs


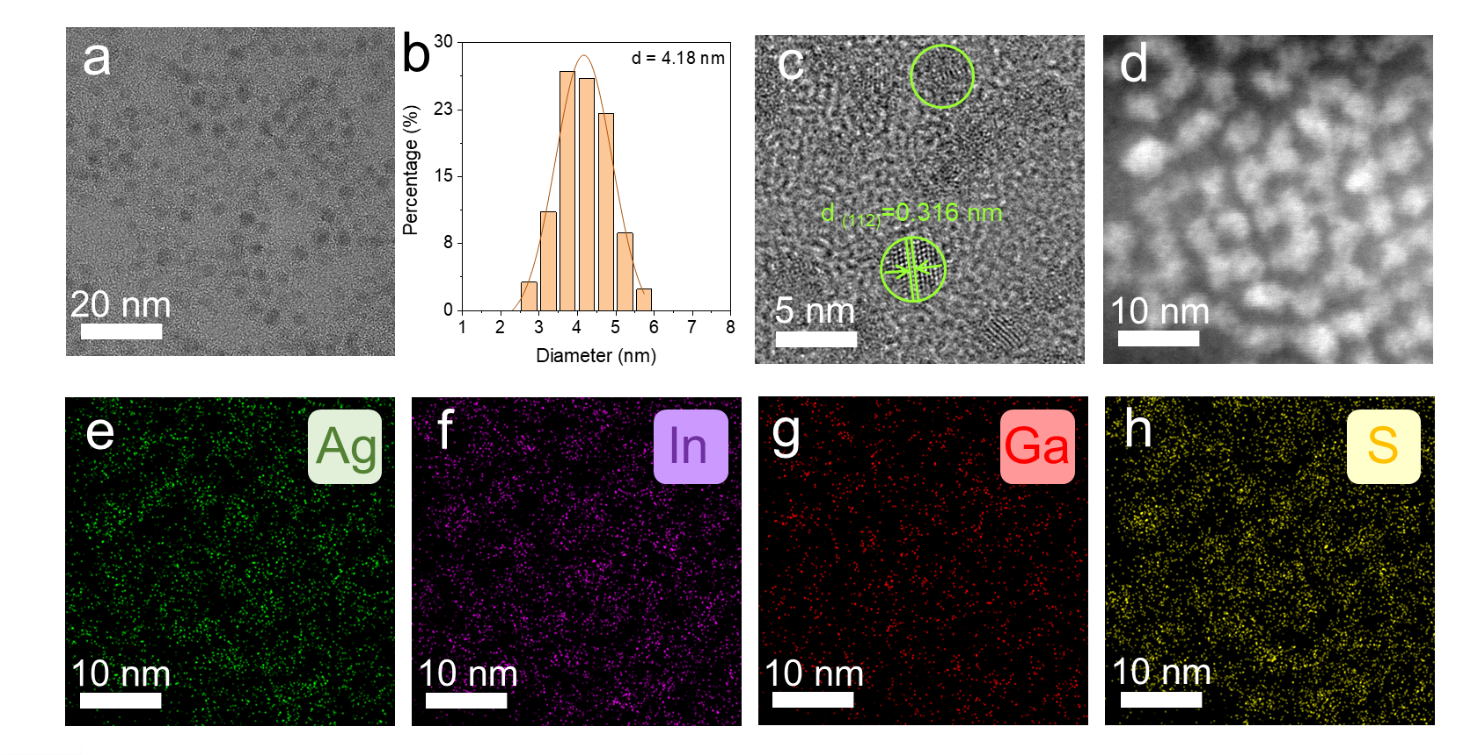


**Fig. S6** (**a**) TEM image, (**b**) Size distribution histogram, (**c**) HRTEM image of AIGS QDs before GS coating. (**d-h**) energy dispersive spectroscopy elemental maps of Ag/In/Ga/S of AIGS QDs before GS coating


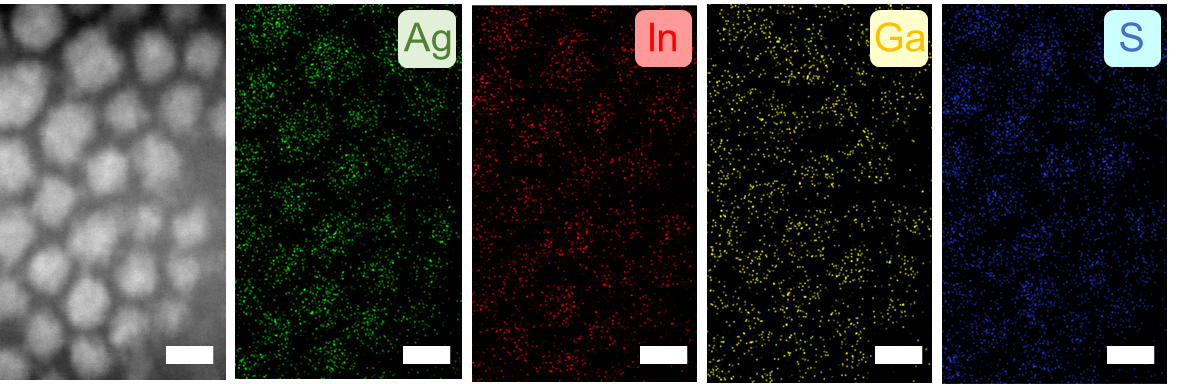


**Fig. S7** Energy dispersive spectroscopy elemental maps of Ag/In/Ga/S of control AIGS QDs, the scale bar is 5 nm


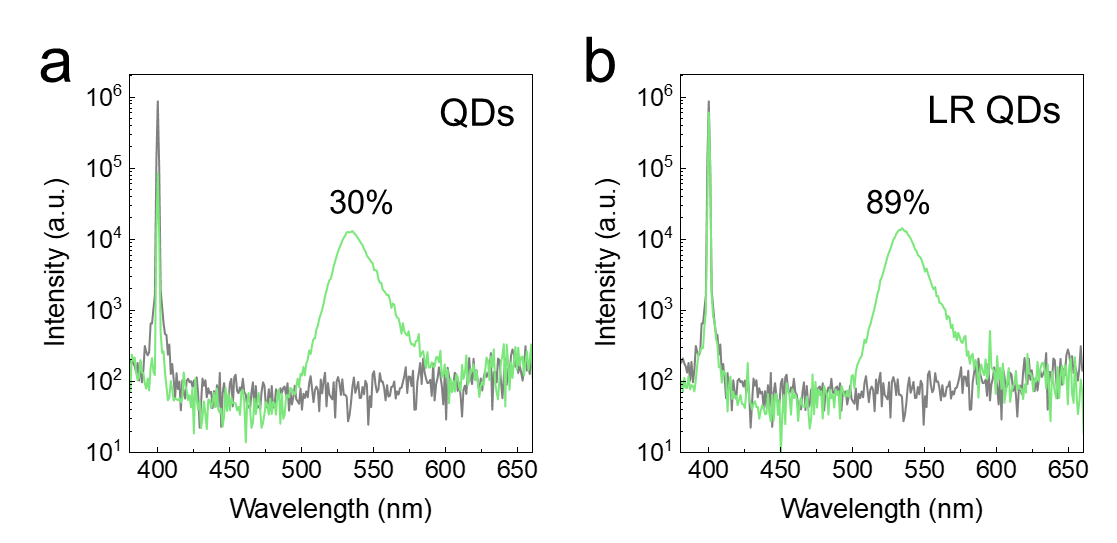


**Fig. S8** PLQY spectra of (**a**) control QDs and (**b**) LR QDs


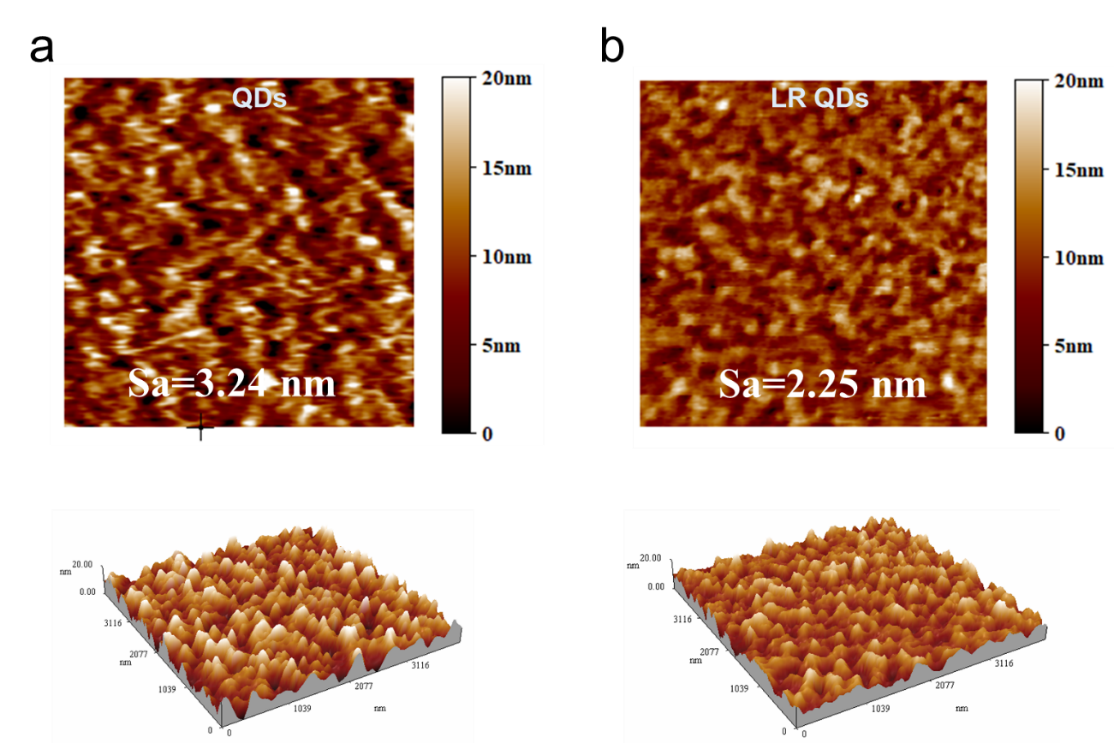


**Fig. S9** AFM image and roughness of (**a**) control QD film and (**b**) LR QD film


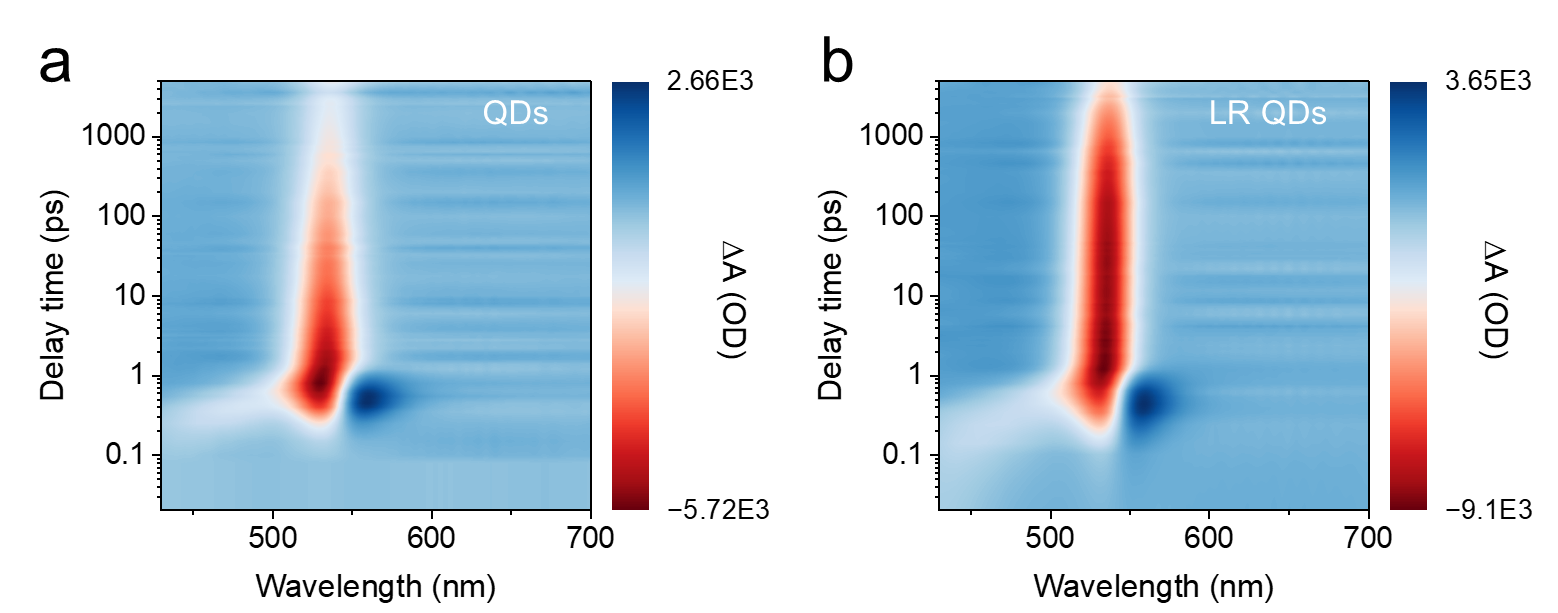


**Fig. S10** The fs-TA spectra of (**a**) control QDs and (**b**) LR QDs


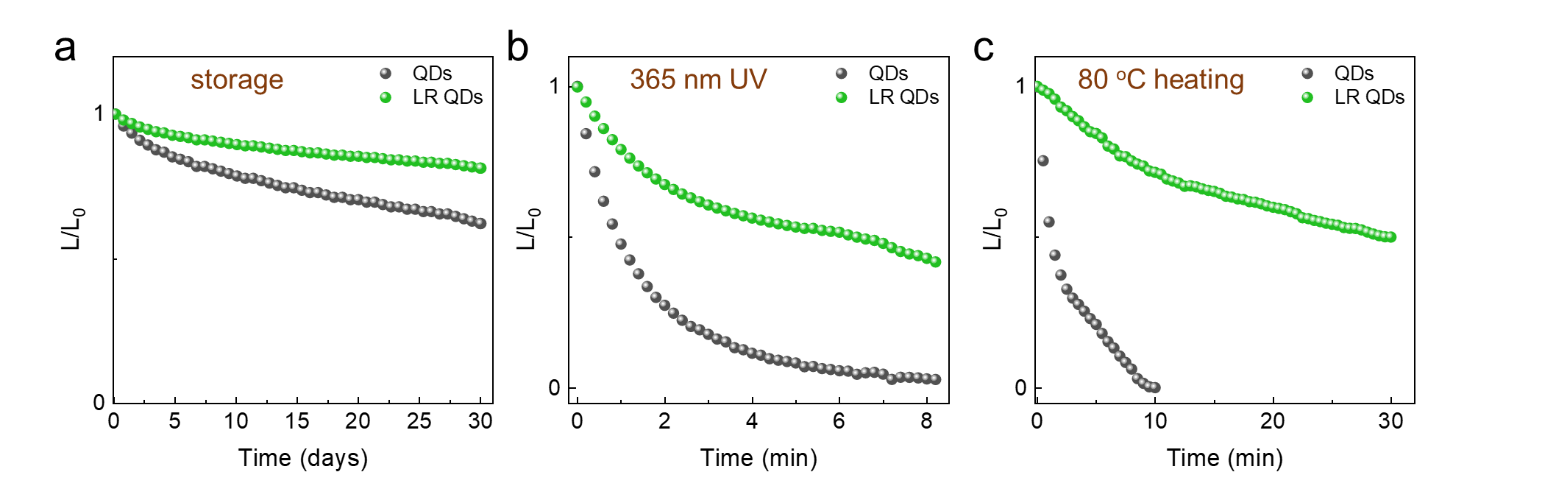


**Fig. S11** (**a**) The PL attenuation curves of colloidal QDs at atmosphere for one month. PL attenuation of QD film (**b**) under 365 nm UV light irradiation and (**c**) heating at 80 °C


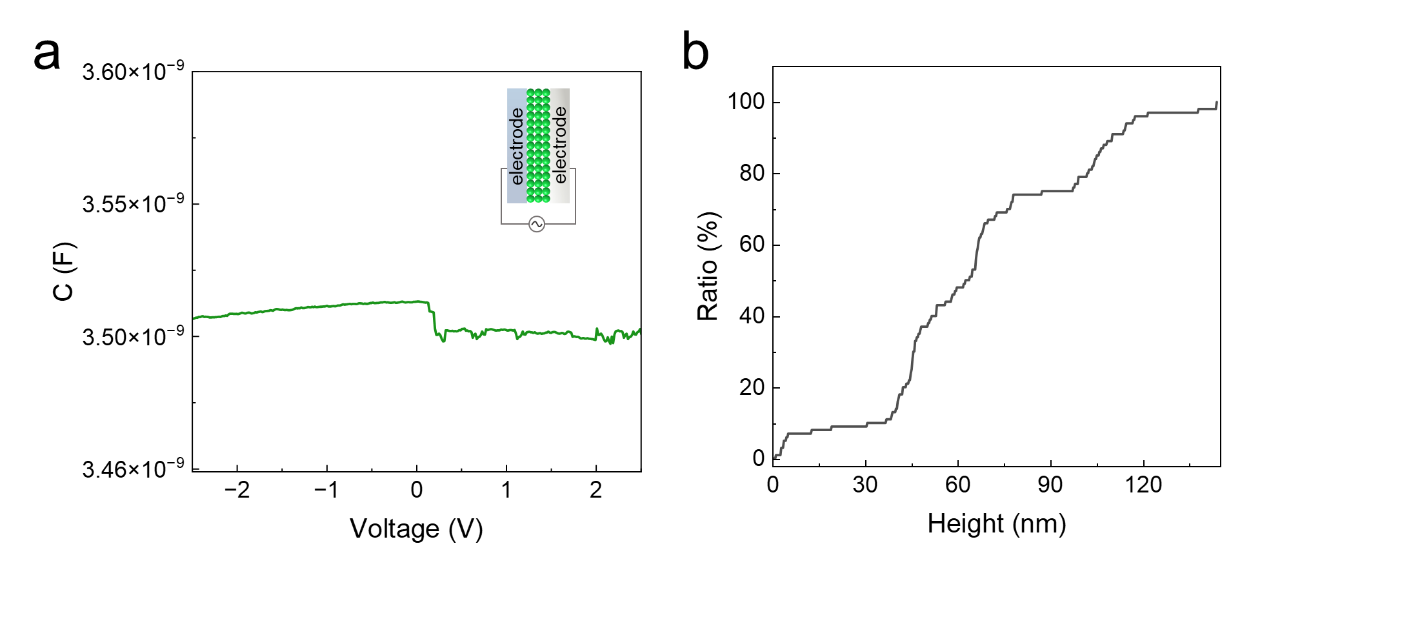


**Fig. S12** (**a**) *C-V* curve of ITO/QDs/Al tested at 700 KHz. (**b**) AFM line scanning of QD film

The relative dielectric constant (*ε*) of AIGS/GS QDs are tested using a flat capacitor with the structure of ITO/QDs/Al, and calculated its relative dielectric constant using the formula $\varepsilon=\frac{Cd}{\varepsilon_{0}A}$. *C* denotes the maximum capacitance achieved under stable conditions (Fig. S12a); *d* represents the thickness of the tested sample, which was determined to be approximately 140 nm via AFM measurement (Fig. S12b); A represents the effective electrode area (6 mm^2^), *ε_0_* represents the vacuum dielectric constant (8.85×10^-12^ F/m). Ultimately, the dielectric constant of the QDs, as experimentally determined, was approximately 9.3.


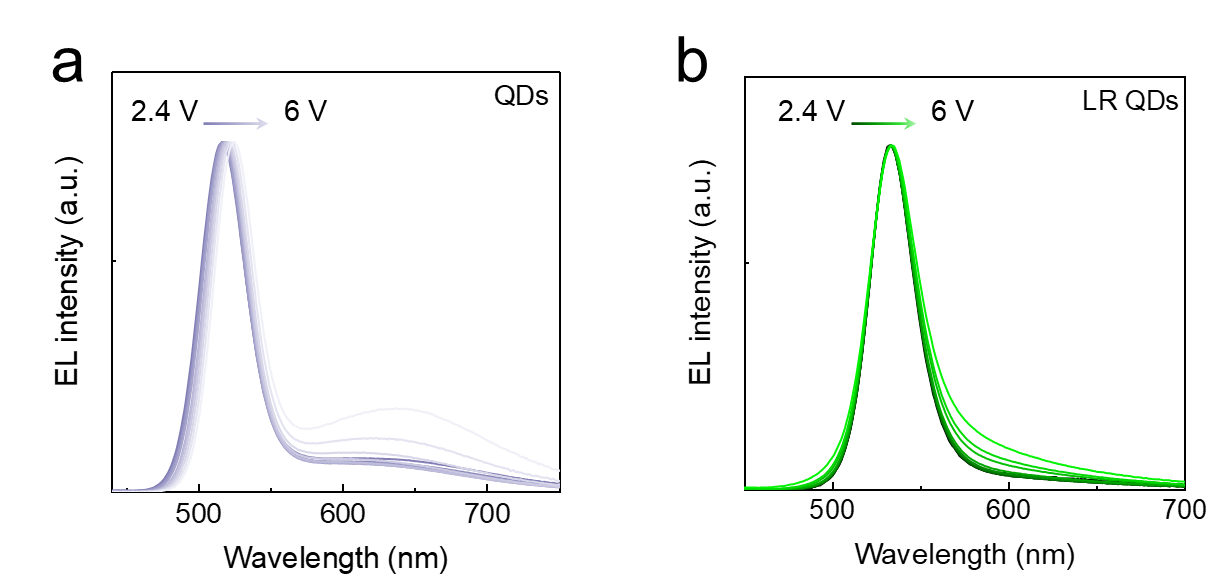


**Fig. S13** EL spectra of (**a**) control QLEDs and (**b**) LR QLEDs tested from 2.4 V to 6 V


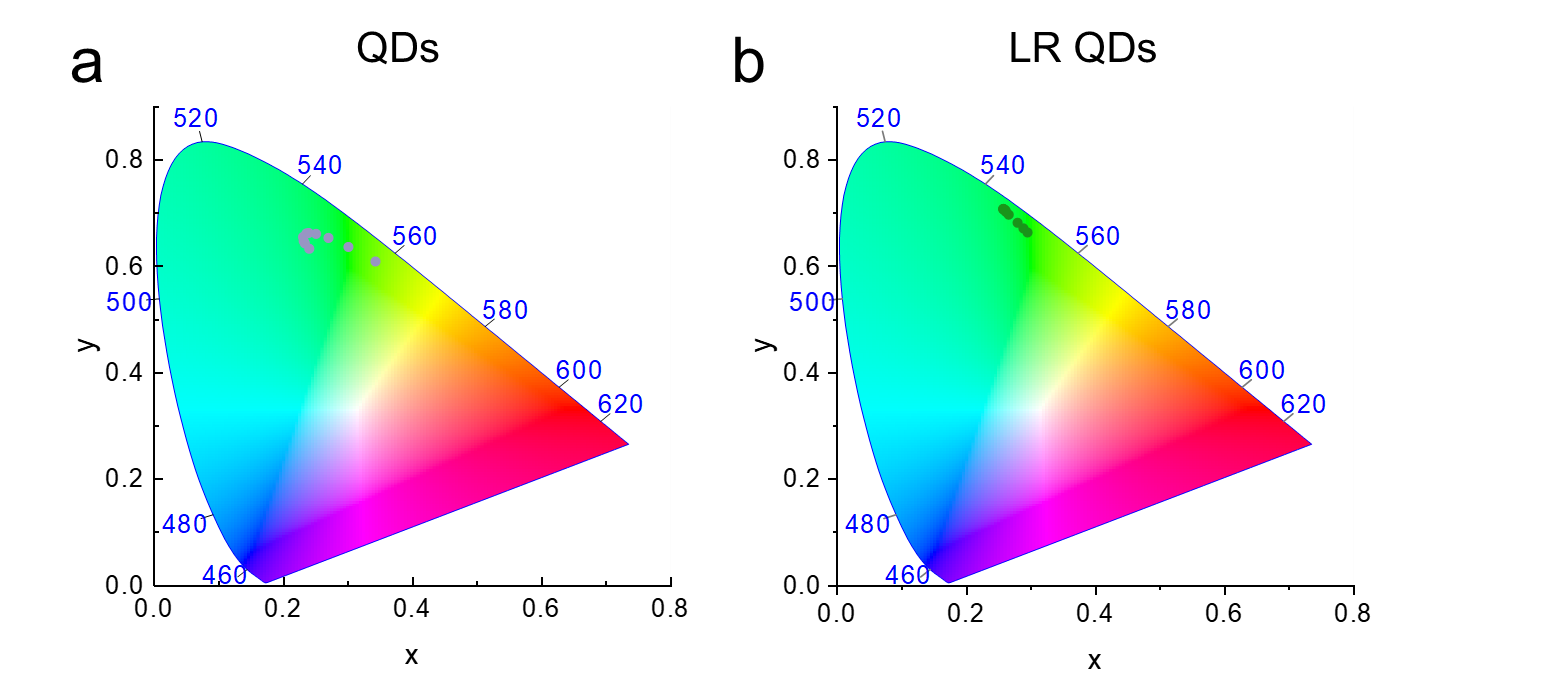


**Fig. S14** The CIE chromaticity coordinates of (**a**) control and (**b**) LR QLEDs


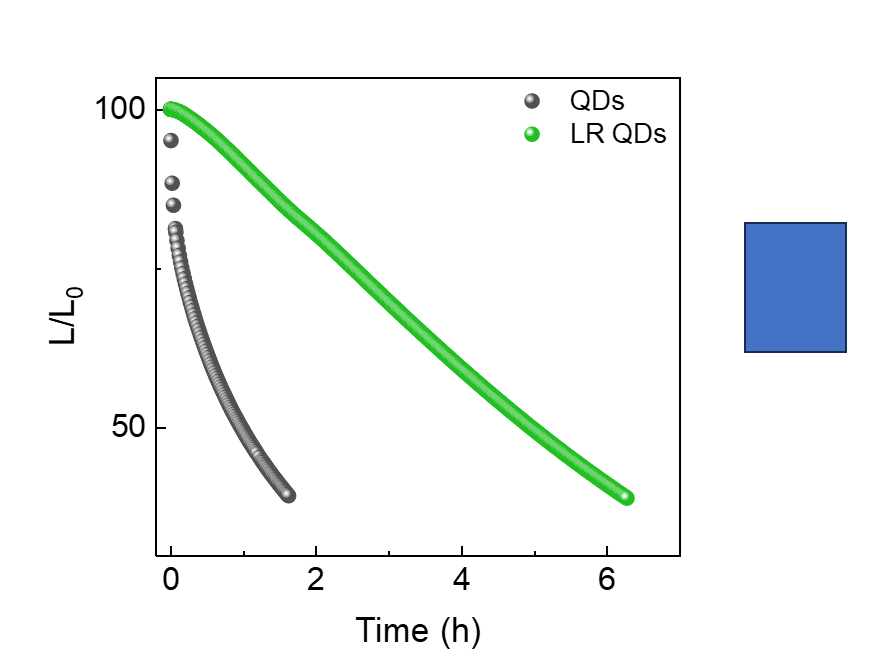


**Fig. S15** The operational lifetime of QLEDs tested at initial luminance of 100 cd/m^2^

**Table S1** The atomic ratio of elemental from ICP characterization

|  | **QDs** | **LR QDs** |
| --- | --- | --- |
| **Ag** | 2.5 | 2.6 |
| **In** | 0.6 | 0.7 |
| **Ga** | 1.6 | 1.8 |
| **S** | 4.1 | 4.5 |
| **ratio** | **1:0.24:0.64:1.64** | **1:0.26:0.69:1.73** |

**Table S2** Summary of TR-PL decay fitting parameters for control and LR QDs

|  | τ_1_ (ns) | **α_1_** | τ_2_ (ns) | **α_2_** | τ_3_ (ns) | **α_3_** | τ_ave_ (ns) |
| --- | --- | --- | --- | --- | --- | --- | --- |
| LR QDs | 18.9 | 17.5% | 67.5 | 65.2% | 273.8 | 17.3% | 94.6 |
| QDs | 12.2 | 31% | 64.6 | 43.2% | 289.3 | 25.8% | 106 |

**Table S3** Summary of GSB fitting parameters for control and LR QDs

|  | τ_1_ (ps) | **α_1_** | τ_2_ (ps) | **α_2_** | τ_3_ (ps) | **α_3_** | τ_ave_ (ps) |
| --- | --- | --- | --- | --- | --- | --- | --- |
| LR QDs | 2.47 | 0.02% | 5099.2 | 99.18% | 308.8 | 0.8% | 94.6 |
| QDs | 1.67 | 0.18% | 64 | 3.16% | 2229.5 | 96.66% | 106 |

**Table S4** Performance summary of reported AIGS QLEDs

| **Time** | **Material** | **PL**  **(nm)** | **FWHM**  **(nm)** | **PLQY**  **(%)** | **EQE**  **(%)** | **Luminance**  **(cd/m^2^)** | **References** |
| --- | --- | --- | --- | --- | --- | --- | --- |
| This work | AIGS@GS | 531 | 31 | 89 | 8.4 | 1219 |  |
| 2024 | AIGS@GS | 524 | 30 | 53 | 5.4 | >1000 | *Adv. Phys. Res., 2024, 3, 240042* |
| 2024 | AIGS@AGS | 532 | 36 | 45 | 0.75 | 2747 | *J. Mater. Chem. C, 2024, 12, 6528* |
| 2024 | AIGS@AGS | 532 | 33 | 45 | 0.26 | 1518 | *Chinese J. Lumin., 2024, 45, 1849* |
| 2024 | AIGZS | 482, 532, 620 | 49, 38, 44 |  | 0.53, 0.13, 0.03 | 405, 184, 196 | *Nano Lett., 2024, 24, 9683* |
| 2024 | AIGS@ZS | 553 | 44.6 | 16 | 0.1 |  | *Chem. Commun., 2024, 5731* |
| 2023 | AIGS@GS | 531 | 32 | 32.0 | 1.10 | 175 | *ACS Appl. Mater. Interfaces., 2023, 15, 8336* |
| 2023 | ZAIGS@IS | 628 | 78 | 86.2 | 5.32 | 218.1 | *ACS Appl. Mater. Interfaces., 2023, 15, 50254* |
| 2022 | AGZS | 470 | 48 | 16.7 | 0.40 | 123.1 | *J. Phys. Chem. Lett., 2022, 13, 11857* |
| 2020 | AIS@ZS | 666 | 122 | 72.0 | 1.25 | 1120 | *J. Phys. Commun., 2020, 4, 045016* |
| 2020 | AIS@GS | 560 | 45 | 70.0 | 0.54 | 120.5 | *Appl. Phys. Lett., 2020, 117, 091101* |

**Supplementary References**

1. P. Hohenberg, W. Kohn, Inhomogeneous electron gas. Phys. Rev. **136**(3B), B864 (1964). <https://doi.org/10.1103/PhysRev.136.B864>
2. W. Kohn, L.J. Sham, Self-consistent equations including exchange and correlation effects. Phys. Rev. **140**(4A), A1133 (1965). <https://doi.org/10.1103/PhysRev.140.A1133>
3. J.P. Perdew, K. Burke, M. Ernzerhof, Generalized gradient approximation made simple. Phys. Rev. Lett. **77**(18), 3865 (1996). <https://doi.org/10.1103/PhysRevLett.77.3865>
